# Complete Genome Sequence of a Novel Variant of Wenzhou Mammarenavirus

**DOI:** 10.1128/genomeA.01303-17

**Published:** 2017-11-22

**Authors:** Meng-meng Liu, Li-li Li, Xiao-feng Wang, Zhao-jun Duan

**Affiliations:** a Department of Medical Microbiology, Qingdao University Medical College, Qingdao, China; b National Institute for Viral Disease Control and Prevention, China CDC, Beijing, China

## Abstract

Wenzhou mammarenavirus (WENV) is a novel species of *Arenavirus* that was first detected in China. We report here a novel WENV variant (WENV G107) from liver samples of *Rattus norvegicus* that shares 84.7% to 86.8% (large [L] segment) and 84.2% to 87.8% (small [S] segment) nucleotide sequence identity with its closest relative. The genomic characteristics were analyzed based on the whole genome of WENV G107.

## GENOME ANNOUNCEMENT

The family *Arenaviridae* includes two genera, *Mammarenavirus* and *Reptarenavirus* (1). *Mammarenavirus* is divided into the Old World and New World arenaviruses. Wenzhou mammarenavirus (WENV) is a single-stranded RNA virus and comprises a new species within the Old World arenaviruses (2). The WENV IgG of the Cardamones variant has been tested against lymphocytic choriomeningitis virus (LCMV)-positive human sera by enzyme-linked immunosorbent assay (ELISA), and *Arenavirus* RNA has been detected in respiratory specimens of Cambodian patients by reverse transcription-PCR (RT-PCR), albeit at a low rate (0.6%) (3).

We describe here the full-length sequence and genome organization of a novel WENV variant. Phylogenetic analysis based on the nucleotide sequences of the L and S segments revealed that this strain clusters with other known WENVs but forms an independent clade.

WENV consists of two segments, large (L) and small (S). The L segment is 7,131 nucleotides (nt) in length; the 5′ end comprises the 52-nt untranslated region (UTR), and the 3′ end contacts the 8-nt UTR. It includes two open reading frames (ORFs) encoding a zinc finger-like protein of 91 amino acids (aa) and a viral RNA-dependent RNA polymerase (RdRp) of 2,229 amino acids (aa) with a 108-nt intergenic noncoding region between the two ORFs. The S segment is 3,322 nt with a 48-nt UTR at the 5′ terminus and a 29-nt UTR at the 3′ terminus. It also includes two ORFs encoding a glycoprotein precursor of 492 aa and a nucleocapsid (NP) of 567 aa with a 62-nt intergenic noncoding region between the two ORFs. RdRp and NP are encoded from the reverse complement sequence of the L and S mRNAs. WENV G107 shares 84.7% to 86.8% (L segment) and 84.2% to 87.8% (S segment) nucleotide sequence identity with its closest relative.

The intergenic noncoding region (IGR) is predicted to fold single or double stem-loop structures in other arenaviruses (4, 5). The secondary structure of IGR within the S segment was predicted using RNAfold and a Web server; it includes one stem-loop in the WENV G107 sequence and in four known WENVs (GenBank accession numbers KJ909794, KM386660, KC669696, and KC669694). Our stem-loop structure consists of 15 G-Cs and 4 A-Us, but the other four WENV strains contain 15 G-Cs and 5 A-Us.

A previous report revealed UTRs in the 5′ and 3′ ends of the L and S segments that exhibit terminal complementarity and a high degree of sequence conservation (6). However, these features are not present in the WENV G107 sequence. In both segments, the 5′ end has no cap structure but includes six nontemplated G bases, and the 3′ end has no poly(A) tail.

### Accession number(s).

The WENV G107 sequence was deposited in GenBank under accession numbers MF925714 (segment L) and MF925715 (segment S).
